# Phage-Encoded LuxR-Type Receptors Responsive to Host-Produced Bacterial Quorum-Sensing Autoinducers

**DOI:** 10.1128/mBio.00638-19

**Published:** 2019-04-09

**Authors:** Justin E. Silpe, Bonnie L. Bassler

**Affiliations:** a Department of Molecular Biology, Princeton University, Princeton, New Jersey, USA; b Howard Hughes Medical Institute, Chevy Chase, Maryland, USA; Max Planck Institute for Terrestrial Microbiology; NCI-NIH; University of Pittsburgh

**Keywords:** LuxR, phage, acyl homoserine lactone, autoinducer, lysis, lysogeny, quorum sensing

## Abstract

Quorum sensing (QS) is a process of cell-to-cell communication that bacteria use to orchestrate collective behaviors. QS relies on the cell-density-dependent production, accumulation, and receptor-mediated detection of extracellular signaling molecules called autoinducers (AIs). Gram-negative bacteria commonly use *N*-acyl homoserine lactones (AHLs) as their AIs, and they are detected by LuxR-type receptors. Often, LuxR-type receptors are insoluble when not bound to a cognate AI. In this report, we show that LuxR-type receptors are encoded on phage genomes, and in the cases we tested, the phage LuxR-type receptors bind to and are solubilized specifically by the AHL AI produced by the host bacterium. We do not yet know the viral activities that are controlled by these phage QS receptors; however, our observations, coupled with recent reports, suggest that their occurrence is more widespread than previously appreciated. Using receptor-mediated detection of QS AIs could enable phages to garner information concerning the population density status of their bacterial hosts. We speculate that such information can be exploited by phages to optimize the timing of execution of particular steps in viral infection.

## Abstract (precis)

Bacteria communicate with chemical signal molecules to regulate group behaviors in a process called quorum sensing (QS). In this report, we find that genes encoding receptors for Gram-negative bacterial QS communication molecules are present on genomes of viruses that infect these bacteria. These viruses are called phages. We show that two phage-encoded receptors, like their bacterial counterparts, bind to the communication molecule produced by the host bacterium, suggesting that phages can “listen in” on their bacterial hosts. Interfering with bacterial QS and using phages to kill pathogenic bacteria represent attractive possibilities for development of new antimicrobials to combat pathogens that are resistant to traditional antibiotics. Our findings of interactions between phages and QS bacteria need consideration as new antimicrobial therapies are developed.

## OBSERVATION

Bacteria engage in the cell-cell communication process called quorum sensing (QS) to coordinate group behaviors. QS relies on production, release, and group-wide detection of extracellular signal molecules called autoinducers (AIs). QS-mediated communication systems are now known to be common in the bacterial domain (1). Growing evidence suggests that viruses of bacteria, called phages, also possess communication capabilities, an idea that originated when Hargreaves et al. reported the presence of genes resembling those encoding Gram-positive bacterial QS components (*agr*) on a phage infecting Clostridium difficile (2). More-recent studies show that phages can engage in their own QS-like dialogs to promote population-wide lysogeny (3), and phages can eavesdrop on bacterial QS-mediated communication to promote population-wide lysis (4). In the eavesdropping case, which is most pertinent to the observations we report here, a vibriophage encodes a homolog of a *Vibrio* QS receptor/transcription factor called VqmA. Host-encoded VqmA and phage-encoded VqmA (VqmA_Phage_) both bind the same AI, 3,5-dimethylpyrazin-2-ol (DPO). In the case of the host, binding of DPO by VqmA launches the QS group behavior program (5). In the case of the phage, binding of DPO by VqmA_Phage_ activates the lysis program, and the phage kills the host. Thus, the vibriophage lysis-lysogeny decision is linked to the population density status of the host cells (4).

**FIG 1:**
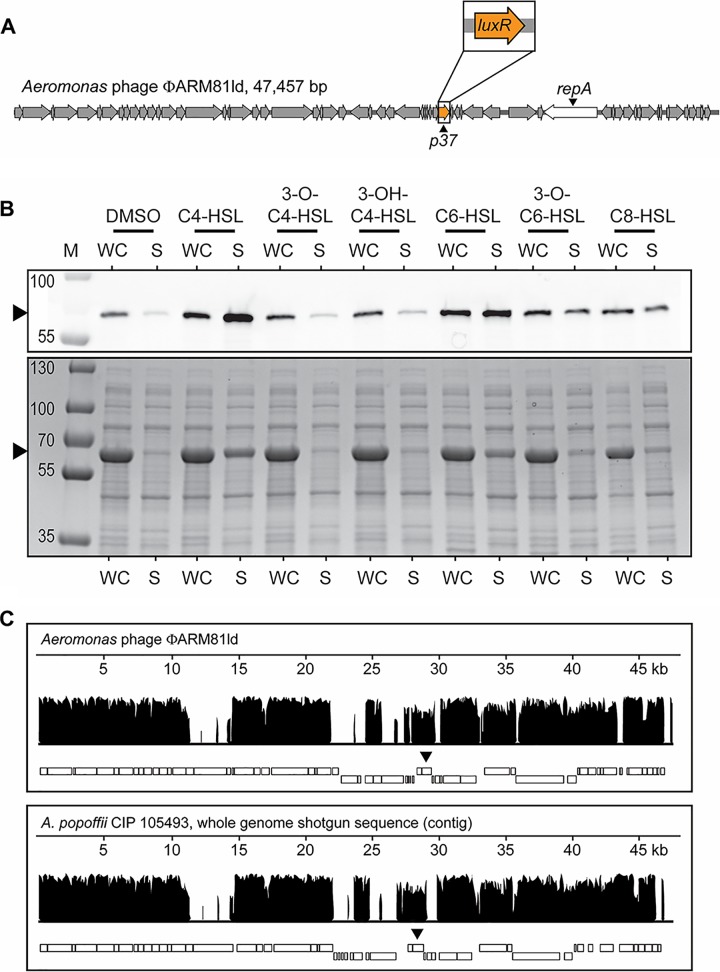
Phage ΦARM81ld encodes a QS LuxR-type receptor that is solubilized by bacterial AIs, and *A. popoffii* is lysogenized by a phage-like element similar to ΦARM81ld. (A) Genome organization of phage ΦARM81ld. *p37* is predicted to encode a LuxR-type receptor (orange). The phage replication gene *repA* is displayed in white. (B) Western blot (top) and total protein (bottom) showing HALO-LuxR_ΦARM81ld_ in the whole-cell (WC) lysate and soluble (S) fractions of recombinant E. coli supplied with 75 µM of the indicated AHL or an equivalent volume of DMSO. The black arrowheads indicate the positions of HALO-LuxR_ΦARM81ld_. M denotes marker (PageRuler Plus; representative bands are labeled). Regarding the differences in band intensities, Coomassie stains total protein, folded and unfolded, whereas the HALO-Western technique requires that the HALO tag be functional for detection. Thus, the HALO-LuxR_ΦARM81ld_ bands in the WC samples show only the fraction of the protein that is folded and functional, and thus, they appear fainter than in the corresponding bands in the S fractions that show all of the folded and functional protein. Consistent with this interpretation, the Coomassie-stained gel shows that there is indeed more total HALO-LuxR_ΦARM81ld_ present in the WC fractions than in the S fractions. (C) Whole-phage-genome alignment of the known phage ΦARM81ld against the full-length *A. popoffii* phage-like contig. Nucleotide sequence identity is shown on the vertical axes. The lengths of the aligned genomes are shown on the horizontal axes. The rectangles below each plot represent predicted ORFs. The upper and lower boxes represent the plus and minus strands, respectively, on which the ORFs are encoded. The black arrowheads indicate the locations of the phage *luxR* genes. Alignment was performed using the progressive Mauve algorithm (see Methods).

Inspired by the above findings, we wondered whether additional phage-bacterium QS connections could exist. To explore this possibility, we performed database analyses scanning for QS receptors encoded by viral genomes. We focused on genes encoding *N*-acyl homoserine lactone (AHL)-binding LuxR-type receptors/transcription factors because they exist in thousands of sequenced genomes of Gram-negative bacteria, making them an easily recognizable type of QS AI-receptor pair. Our bioinformatic search for receptors belonging to the “transcription factor LuxR-like, AI-binding domain” superfamily of proteins (InterPro IPR036693) revealed 12,285 AHL-binding LuxR domain entries encoded by bacteria and two additional entries in a category outside living organisms, among viruses. One hit was present on a putative *Myoviridae* phage (GenBank accession no. MH622937.1) in metagenomic data from animal viruses (Christopher Buck, NIH, personal communication; see Fig. S1A in the supplemental material). Because this DNA sequence is not a verified phage and the phage does not exist in a known or available host bacterial strain, we could not pursue this finding further. The second viral *luxR*-type gene was on a characterized phage that infects *Aeromonas* sp. strain ARM81 (accession no. KT898133.1). This phage is called ΦARM81ld (6) (Fig. 1A), and the gene designated *p37* encodes a putative LuxR-type transcription factor. While we were unable to obtain *Aeromonas* sp. ARM81 or the resident phage, we synthesized the *p37* gene and cloned it into a recombinant expression vector. Hereafter, we call the protein encoded by *p37* LuxR_ΦARM81ld_.

10.1128/mBio.00638-19.1 FIG S1 The uncharacterized *Myoviridae* phage encodes a LuxI-LuxR pair, and *A. popoffii* encodes multiple LuxR-type QS receptors. (A) Genome organization of the uncharacterized *Myoviridae* phage predicted to encode *luxI* and *luxR* genes (blue and orange, respectively). (B) Amino acid sequence alignment of LuxR_ΦARM81ld_ with LuxR homologs from *A. popoffii* and the characterized AhyR QS receptor of *A. hydrophila*. *A. popoffii* homologs 1 and 2 represent the host LuxR receptor and the phage-encoded LuxR_Apop_ receptor, respectively. Sites of high, low, and no identity are shaded black, gray, and white, respectively. Residues are numbered according to their positions in the consensus sequence. Download FIG S1, TIF file, 2.2 MB. Copyright © 2019 Silpe and Bassler. 2019 Silpe and Bassler This content is distributed under the terms of the Creative Commons Attribution 4.0 International license.

Commonly, bacterial LuxR-type receptors require their cognate AHL ligands to fold and, in turn, become soluble (7). To determine whether the phage-encoded LuxR_ΦARM81ld_ receptor follows the same constraints, we expressed HALO-tagged LuxR_ΦARM81ld_ in Escherichia coli. SDS-PAGE and Western blotting with HALO-Alexa_660_ showed that the LuxR_ΦARM81ld_ protein was produced and present in the whole-cell lysate, but not in the soluble fraction (Fig. 1B). We reasoned that LuxR_ΦARM81ld_ might bind the C4-homoserine lactone (C4-HSL) AI produced by members of the host *Aeromonas* genus (8). Indeed, Fig. 1B shows that the addition of C4-HSL to the E. coli carrying HALO-LuxR_ΦARM81ld_ enabled the HALO-LuxR_ΦARM81ld_ to fold and become soluble, as it was present in both the whole-cell lysate and the soluble fraction. To examine ligand specificity, we assessed the ability of a panel of AHL AIs with various tail lengths (C4, C6, and C8) and decorations (3-OH and 3-O) to solubilize LuxR_ΦARM81ld_. LuxR_ΦARM81ld_ is solubilized by C4-HSL, but not by AHLs with C4 tails containing decorations (Fig. 1B). AHLs containing acyl tails longer than C4 or a longer tail and a decoration (3-O-C6-HSL) display diminished abilities to solubilize LuxR_ΦARM81ld_ compared to C4-HSL (Fig. 1B). These results demonstrate that, like bacterial LuxR-type receptors which often show exquisite specificity for their cognate AHL AIs, the phage-encoded LuxR_ΦARM81ld_ receptor is solubilized primarily by the AHL AI reportedly produced by its *Aeromonad* host genus.

There are no homologs identical to LuxR_ΦARM81ld_ in the NCBI database; however, LuxR_ΦARM81ld_ shares ∼48% identity with the bacterial QS receptor encoded by the human pathogen Aeromonas hydrophila called AhyR (8) and ∼60% homology to a LuxR-type receptor on a DNA contig from an uncharacterized isolate of Aeromonas popoffii CIP 105493 (9). Curiously, A. popoffii CIP 105493 has at least two genes encoding putative LuxR receptors (Fig. S1B). The first *A. popoffii* homolog, with 48% identity to LuxR_ΦARM81ld_, shares 95% identity with A. hydrophila AhyR and is located in the same genomic context as *ahyR* in *A. hydrophila* (neighboring a putative *luxI* gene called *ahyI*), indicating that this first homolog is likely the *A. popoffii* bacterial QS receptor. The second *A. popoffii* homolog has less than 50% identity to *A. hydrophila* AhyR but, as mentioned, shares ∼60% identity to LuxR_ΦARM81ld_ (Fig. S1B). This gene is located on an ∼47-kb contig that, by PHASTER analysis (10), is predicted to be an intact phage, suggesting that like LuxR_ΦARM81ld_, this second *A. popoffii luxR* gene exists on a phage. Consistent with this idea, we found that, as is the case for *Aeromonas* sp. ARM81 (6), lysis of *A. popoffii* is induced by the DNA damaging agent mitomycin C (MMC). Specifically, a precipitous decline in OD_600_ over time occurs after the addition of MMC (Fig. S2A). DNA specific to the phage-like contig can be amplified from the clarified supernatants of DNase-treated culture lysates, suggesting that phage particles are present. Furthermore, PCR amplification of the putative phage origin and primase gene required for replication (*repA*), ligation of the product to an antibiotic resistance cassette, followed by introduction and selection in E. coli, show that the ligated product can be maintained as a plasmid. This final result demonstrates that the *luxR*-containing contig encodes a functional replication gene for a plasmid-like element, which is the reported state in which ΦARM81ld exists when it lysogenizes its *Aeromonas* host (6). Lastly, the contig carrying this putative phage *luxR* gene is comparable in length to the genome of phage ΦARM81ld (46.8 kb in *A. popoffii* versus 47.6 kb in ΦARM81ld) and can be aligned along its entire length to the complete ΦARM81Id genome (60.1% pairwise identify; Fig. 1C). Thus, we hypothesize that *A. popoffii* harbors an extrachromosomally replicating prophage encoding a *luxR* QS gene similar to that of ΦARM81ld. We hereafter refer to the phage in *A. popoffii* as Apop and the phage LuxR receptor as LuxR_Apop_. Similar to the vibriophage-*Vibrio* case described above (4), because of the low sequence identity, the QS receptors encoded on ΦARM81ld and on Apop do not appear to be the result of direct transfer from the bacterial host.

10.1128/mBio.00638-19.2 FIG S2 MMC induces lysis of *A. popoffii*, and host LuxR-type receptors are more similar to each other than to phage LuxR-type receptors. (A) Growth curve of *A. popoffii* strain CIP 105493 in the absence (black) or presence (white) of 200 ng ml^−1^ MMC. Data are represented as means ± standard deviations for four biological replicates. (B) Amino acid sequence alignment of the *Aeromonas* sp. ARM81 and *A. popoffii* LuxR-type receptors. (C) Amino acid sequence alignment of the phage ΦARM81ld and Apop LuxR-type receptors. In panels B and C, black and gray shading show identical and similar residues, respectively. Residues are numbered according to their positions in the consensus sequence. Download FIG S2, TIF file, 1.9 MB. Copyright © 2019 Silpe and Bassler. 2019 Silpe and Bassler This content is distributed under the terms of the Creative Commons Attribution 4.0 International license.

**FIG 2:**
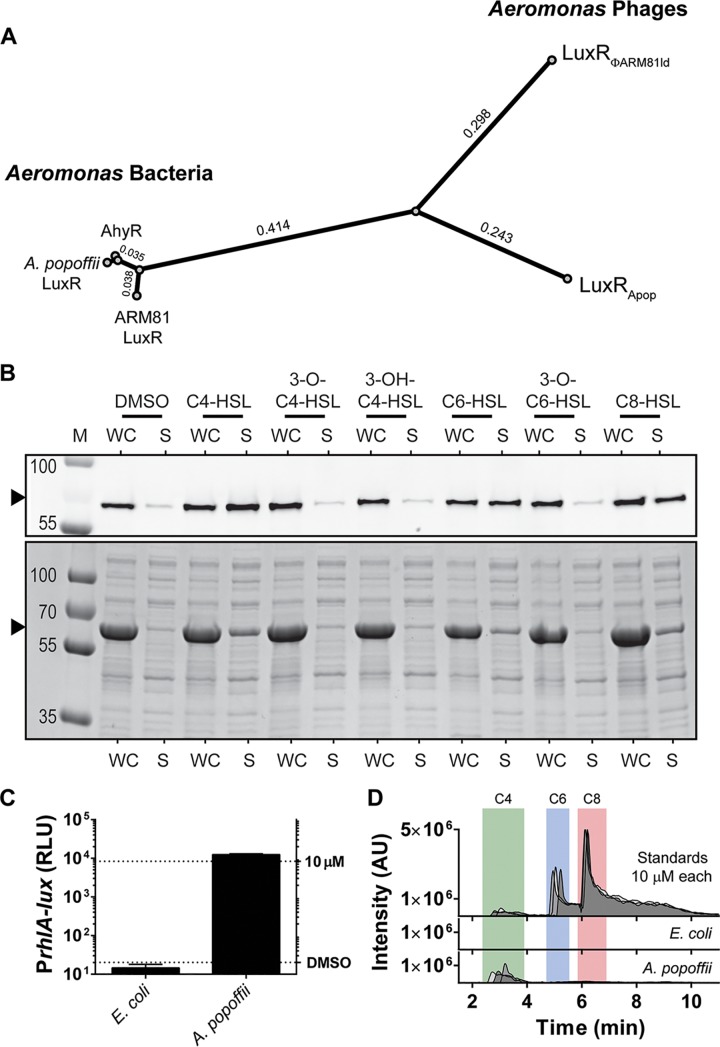
Host and phage harbor distinct LuxR proteins, and the phage LuxR_Apop_ protein is solubilized by the AI produced by its host. (A) Phylogenetic analysis of three bacterially encoded LuxR proteins (AhyR from *A. hydrophila* and the LuxR-type receptors from *Aeromonas* sp. strain ARM81 and *A. popoffii*) and the phage-encoded LuxR-type receptors (LuxR_ΦARM81ld_ and LuxR_Apop_). Branch lengths are indicated. (B) Western blot (top) and total protein (bottom) showing HALO-LuxR_Apop_ in the whole-cell (WC) lysate and soluble (S) fractions from recombinant E. coli supplied with 75 µM of the indicated AHL or an equivalent volume of DMSO. The black arrowheads indicate the positions of HALO-LuxR_Apop_. M denotes marker (PageRuler Plus; representative bands are labeled). (C) Bioassay to detect C4-HSL based on the Pseudomonas aeruginosa RhlR receptor and the target *rhlA* promoter. The E. coli bioassay strain does not produce C4-HSL. Thus, P*rhlA-lux* expression depends on exogenous C4-HSL. Shown is P*rhlA-lux* activity following the addition of 25% (vol/vol) of cell-free culture fluids prepared from the indicated strains. Bioluminescence is shown in relative light units (RLU). Data are represented as means plus standard deviations (error bars) for three biological replicates. The top and bottom dotted lines designate the reporter activity in response to 10 µM C4-HSL and after the addition of an equivalent volume of DMSO solvent, respectively. (D) LC-MS chromatogram of 10 µM C4-HSL (green), C6-HSL (blue), and C8-HSL (red) standards in filtered LB medium and the same analyses of cell-free culture fluids prepared from E. coli and *A. popoffii*. Intensity is shown in arbitrary units (AU). The data show three biological replicates for culture fluids and three technical replicates for the standards.

The predicted AhyR-like QS receptors encoded by the *A. popoffii* and *Aeromonas* sp. ARM81 bacterial hosts share more than 90% identity, and yet the corresponding phage-encoded receptors LuxR_Apop_ and LuxR_ΦARM81ld_, respectively, are only ∼60% identical to one another (Fig. 2A; see also Fig. S2B and S2C). This disparity in amino acid identity between the phage LuxR proteins led us to wonder whether the LuxR_Apop_ AI binding preferences were the same or different from those of LuxR_ΦARM81ld_. Of the seven AHLs in our test collection, recombinant LuxR_Apop_ is solubilized by C4-HSL and also by C6- and C8-HSL, but it is not solubilized by AHLs containing tail decorations (Fig. 2B). Consistent with the idea that the two phage receptors recognize the AI produced by their bacterial hosts, cell-free culture fluids from *A. popoffii* activated an E. coli reporter strain that generates light specifically in response to exogenously supplied C4-HSL (Fig. 2C), and LC-MS analysis of the *A. popoffii* culture fluid showed that C4-HSL was present (Fig. 2D) in amounts comparable to those reported for other C4-HSL-producing bacteria (>10 µM by both assays) (11). We could not quantify C6- or C8-HSL in the *A. popoffii* fluids, suggesting that those molecules are either not made by *A. popoffii* or they exist below the limit of our method (∼500 and 250 nM for C6- and C8-HSL, respectively). Taken together, our results suggest that, like ΦARM81ld, the Apop phage encodes a functional LuxR receptor that binds to and is solubilized by C4-HSL, an AI produced by its bacterial host.

We do not yet know what target promoters are controlled by these phage LuxR receptors nor how QS drives phage biology. In the characterized vibriophage-*Vibrio* case, the addition of AI causes lysis when it is bound by the phage QS receptor. We found that addition of C4-, C6-, or C8-HSL to *A. popoffii* does not induce lysis (Fig. S3), and as mentioned, we were unable to obtain the *Aeromonas* sp. ARM81 host strain to test for a similar effect with phage ΦARM81ld. We suspect that, beyond the AI, the phage ΦARM81ld and Apop QS receptor-controlled pathways could require some additional input that is not present in our experiments. Alternatively, the phage QS receptor-controlled pathways could regulate a different aspect of phage-host or phage-phage biology, for example, superinfection exclusion. We are testing such possibilities now.

10.1128/mBio.00638-19.3 FIG S3 Exogenous addition of AHLs to *A. popoffii* does not induce lysis. Growth curve of *A. popoffii* supplemented with the indicated AHL or an equivalent volume of DMSO. Data are represented as means ± standard deviations for four biological replicates. Download FIG S3, TIF file, 1.4 MB. Copyright © 2019 Silpe and Bassler. 2019 Silpe and Bassler This content is distributed under the terms of the Creative Commons Attribution 4.0 International license.

The Apop LuxR-type receptor was revealed through analysis of sequencing data of its host, *A. popoffii*, a bacterium not previously known to harbor a phage. We take this finding as preliminary evidence that additional QS components are encoded on phage genomes and await discovery. It appears that while different members of the same bacterial genus possess highly similar QS receptors, the QS receptors on their resident phages are more varied. Whether this finding hints at general principles underlying evolution of phage-encoded QS receptors can be resolved with the identification of additional examples. Our hypothesis is that the LuxR-type receptors allow the phages to eavesdrop on host bacterial communication and, using the information they glean, the phages optimally alter their biology. This hypothesis mirrors the mechanism described for the vibriophage-*Vibrio* case described above (4). Other phage-bacterium QS interactions are also possible. For example, the uncharacterized *Myoviridae* sp. mentioned above that was identified in our initial search for viral QS receptors, encodes a predicted AHL-binding receptor, and located immediately upstream is an ORF predicted to encode a LuxI-type AI synthase (Fig. S1A). This arrangement suggests a model similar to the phage-phage arbitrium system (3), but with the added complexity that the host could produce and/or respond to the same QS AI as the phage. These occurrences, primarily garnered from database searches, exemplify, as first substantiated by Hargreaves et al., that phage-bacterium QS interactions may be common (2). Future work could be aimed at identifying the regulons controlled by these phage QS components, whether they are phage specific, host specific, or whether they drive both phage and host biology, and their consequences to both the phage and the host.

### Methods. (i) Bacterial strains, plasmids, and growth conditions.

Strains and plasmids used in this study are listed in Tables S1 and S2 in the supplemental material, respectively. E. coli strains and *A. popoffii* CIP 105493 were grown with aeration in Luria-Bertani (LB-Miller, BD-Difco) broth. Unless otherwise indicated, E. coli was grown at 37°C and *A. popoffii* was grown at 30°C. Where appropriate, the following antibiotics and concentrations were used: 100 μg ml^−1^ ampicillin (Amp) (Sigma), 100 μg ml^−1^ kanamycin (Kan) (GoldBio), and 10 μg ml^−1^ chloramphenicol (Cm) (Sigma). The following inducers and concentrations were used: 200 ng ml^−1^ mitomycin C (MMC) (Sigma), 0.1% L-arabinose (Ara) (Sigma), and 200 µM isopropyl β-D-1-thiogalactopyranoside (IPTG) (GoldBio). Unless otherwise indicated, all autoinducers (AIs) were used at 75 μM. WuXi AppTec provided AIs except for 3-OH-C4-HSL, which was made in-house, and C6-HSL, which was obtained from Cayman Chemical.

10.1128/mBio.00638-19.4 TABLE S1 Bacterial strains used in this study. Download Table S1, DOCX file, 0.01 MB. Copyright © 2019 Silpe and Bassler. 2019 Silpe and Bassler This content is distributed under the terms of the Creative Commons Attribution 4.0 International license.

10.1128/mBio.00638-19.5 TABLE S2 Plasmids used in this study. Download Table S2, DOCX file, 0.02 MB. Copyright © 2019 Silpe and Bassler. 2019 Silpe and Bassler This content is distributed under the terms of the Creative Commons Attribution 4.0 International license.

### (ii) Cloning techniques.

Primers used for plasmid construction and for generating the *luxR_ΦARM81ld_* dsDNA gene block sequence are listed in Table S3 (Integrated DNA Technologies). The publicly available annotation of *luxR_ΦARM81ld_* (*p37*) associated with ΦARM81ld (GenBank accession no. KT898133) was used as the reference with the exception that, based on our own sequence alignments, the *luxR_ΦARM81ld_* start codon is more likely 4 codons downstream from the annotated start site.

10.1128/mBio.00638-19.6 TABLE S3 Oligonucleotides and dsDNA used in this study. Download Table S3, DOCX file, 0.02 MB. Copyright © 2019 Silpe and Bassler. 2019 Silpe and Bassler This content is distributed under the terms of the Creative Commons Attribution 4.0 International license.

The T7-based vector for production of the two phage LuxR proteins was constructed so that both proteins had identical C-terminal linkers to a TEV cleavage site and HALO-HIS_6_ tag. The backbone for the expression vectors was prepared by PCR amplification of pJES-178, a vector derived from pH6HTC (catalog no. G8031; Promega Corp.), with primers JSO-1268 × JSO-1435. The amplification strategy generated an N-terminal PacI site and a blunt C-terminal TEV-HALO-HIS_6_ tag. The amplified backbone product was treated with DpnI, CIP, and PacI. The *luxR_ΦARM81ld_* insert was prepared by PCR amplification of JSgblock-93 with JSO-1438 × JSO-1440, followed by treatment with PacI and ligation into the backbone to generate pJES-179. The *luxR_Apop_* insert was prepared by PCR amplification of total DNA extracted from *A. popoffii* CIP 105493 with JSO-1502 × JSO-1503 followed by treatment with PacI and ligation into the backbone to generate pJES-181. The Apop miniplasmid, pJES-180, used to demonstrate the presence of the functional *repA* gene on the phage contig, was constructed by PCR amplification of total DNA extracted from *A. popoffii* with JSO-1452 × JSO-1462 followed by blunt-end ligation with a pRE112-derived PCR fragment (the product of amplification with JSO-931 × JSO-932) encoding Cm^r^ and the *pir*-dependent *oriR6ky* (12). The ligated product was transformed into E. coli TOP10 (Invitrogen), which lacks *pir*, so plasmid replication could be accomplished only via the cloned *repA* gene. The cloned *repA*-containing fragment could be amplified with primers JSO-1522 × JSO-1452.

Q5 high-fidelity polymerase, T4 DNA ligase, and restriction enzymes were obtained from NEB. Constructs were transformed into E. coli TOP10 where they were isolated and verified by sequencing. All DNA was introduced by electroporation using 0.1-cm gap cuvettes (USA Scientific) with a Bio-Rad MicroPulser.

### (iii) Total protein and HALO Western blotting to assess phage LuxR solubility.

The pJES-179 and pJES-181 plasmids carrying HALO-LuxR_ΦARM81ld_ and HALO-LuxR_Apop_, respectively, were transformed into E. coli T7Express *lysY/I^q^* (NEB). Overnight cultures were back-diluted 1:100 into 100 ml fresh medium and grown at 34°C to an OD_600_ of ∼0.4 to 0.6. IPTG was added to the cultures, and subsequently, they were divided into seven 10-ml aliquots. Six of the aliquots received 75 μM of an AI, and the seventh aliquot received an equivalent volume (0.1% vol/vol) of DMSO. The cultures were shaken at 34°C for an additional 8 h. The cells were pelleted at 4,000 rpm for 10 min and stored at −80°C until further use.

For protein solubility assessment, each pellet was thawed, resuspended, and lysed in 200 μl of HALO-BugBuster lysis buffer (1× BugBuster [Millipore] supplemented with 100 mM NaCl, 1 mM DTT, 1× Halt protease inhibitor cocktail [Thermo], 0.5 mM EDTA, 3.5 μM HaloTag-Alexa Fluor 660 ligand [HALO-Alexa_660_; Promega], and 2 μl ml^−1^ lysonase bioprocessing reagent [Millipore]). After 30-min incubation at 30°C, half of each lysate was transferred to a new tube on ice (designated whole-cell lysate fraction [WC]), the remainder was subjected to centrifugation at 15,000 × *g* for 30 min at 4°C, and the clarified supernatant was recovered (designated the soluble fraction [S]). Two microliters of each WC sample was diluted into 28 μl of lysis buffer (15-fold dilution), and 5 μl of each S sample was diluted into 25 μl of lysis buffer (6-fold dilution). Ten microliters of 4× Laemmli sample buffer (Bio-Rad) was added to each tube, followed by incubation at 70°C for 15 to 20 min. Ten microliters of each sample was separated by SDS-PAGE in 4 to 20% Mini-Protein TGX gels (Bio-Rad) and stained with Coomassie R-250 for total protein. A second gel, run in parallel, was loaded with the above WC and S samples diluted a further 15-fold and 6-fold, respectively. HALO-Alexa_660_ was imaged using the Cy5 setting of an ImageQuant LAS 4000 (GE). Exposure times never exceeded 15 s. PageRuler Plus prestained protein ladder (Thermo) was used as the marker.

### (iv) *A. popoffii* growth and lysis assays.

In the *A. popoffii* growth assay, four overnight cultures of *A. popoffii* were back-diluted 1:1,000 into fresh LB broth, and quadruplicate 198-µl aliquots from each culture were dispensed into wells of a 96-well plate containing 2 µl of 10 mM C4-, C6-, or C8-HSL or an equivalent volume of DMSO. In the lysis assay, four overnight cultures of *A. popoffii*, each prepared from a different single colony, were back-diluted 1:100 into LB broth, grown to an OD_600_ of 1.0, and again back-diluted to an OD_600_ of 0.15. From each culture, duplicate 198-µl aliquots were dispensed into wells of a 96-well plate (catalog no. 3904; Corning Costar) containing 2 µl MMC or water. In both the growth and lysis assays, plates were shaken at 30°C, and a BioTek Synergy Neo2 Multi-Mode reader was used to measure OD_600_ every 7.5 min. For the DNase assay, 1 ml of *A. popoffii* culture grown and induced with MMC as described above was allowed to lyse for 5 h at 30°C. The sample was subjected to centrifugation at 15,000 × *g* for 2 min, the supernatant was recovered, and DNase (DNA-free kit; Thermo) was added. After incubation at 37°C for 40 min, followed by DNase inactivation, DNA was purified using the Phage DNA isolation kit (Norgen Biotek). PCR amplification of Apop DNA was carried out using primers JSO-1514 × JSO-1503, and the product was verified by electrophoresis and sequence analysis.

### (v) Bioassay for *A. popoffii*-produced AHLs.

The bioassay to measure C4-HSL in cell-free culture fluids employed a previously published E. coli reporter strain (JP-117) harboring a plasmid with P*rhlA-lux* and a plasmid with pBAD-*rhlR* (13). An overnight culture of JP-117, grown at 30°C, was back-diluted 1:100 in LB broth containing 0.1% L-arabinose and returned to growth at 30°C for 2 h prior to being dispensed into a 96-well plate (150 µl per well). Cell-free culture fluids from an overnight culture of *A. popoffii* or a non-AHL-producing negative control (E. coli T7Express *lysY/I^q^*) were prepared by centrifugation (1 min, 15,000 rpm) followed by filtration through 0.22-μm Costar Spin-X tubes (Corning). Fifty microliters of the resulting cell-free culture fluid was added to each well. For a positive control, a 40 μM synthetic C4-HSL standard was made in LB, and 50 µl of this preparation was added to 150-μl aliquots of the JP-117 reporter strain. Bioluminescence and OD_600_ were measured in a BioTek Synergy Neo2 Multi-Mode reader 7 h later. Relative light units were calculated by dividing the bioluminescence by the OD_600_ of the culture.

### (vi) Liquid chromatography-mass spectrometry to detect C4-, C6-, and C8-HSL.

Cell-free fluids were prepared from overnight cultures of *A. popoffii* or E. coli T7Express *lysY/I^q^* (negative control) as described above. Standards containing synthetic C4-, C6-, and C8-HSL (each at 10 μM) were made in LB broth. Culture fluids and standards were loaded onto a C_18_ column (1 mm by 75 mm) (ACE 3 C18 PFP, Mac-Mod) using a Shimadzu HPLC system and PAL auto-sampler (20 μl per injection) at a flow rate of 70 μl min^−1^, using a previously published method (4). Full scan MS data were acquired with an LTQ-Orbitrap XL mass spectrometer (Thermo) at a resolution of 30,000 in profile mode from the *m/z* range of 170 to 240. C4-, C6-, and C8-HSL were detected by performing XIC at *m/z* of 172.09736 (C_8_H_14_NO_3_), 200.128668 (C_10_H_18_NO_3_), and 228.15996 (C_12_H_22_NO_3_), respectively, each with a mass accuracy of ±10 ppm. Caffeine (2 µM in 50% acetonitrile with 0.1% formic acid) was injected as a lock mass using an HPLC pump (LC Packing) with a flow splitter and delivered at an effective flow rate of 20 µl min^−1^ through a tee at the column outlet. Files were processed in Xcalibur (Thermo).

### (vii) Quantification and statistical analysis.

Data were recorded, analyzed, and plotted using Microsoft Excel and GraphPad Prism 6. Sequencing results, alignments, phylogenetic trees, plasmid maps, and genomes were assembled using SnapGene (GSL Biotech), UGENE (Unipro), Geneious (Biomatters Limited), and ApE (M. Wayne Davis). Whole-genome alignment was performed using Mauve (14). The phage-like contig from *A. popoffii* CIP 105493 and the contig containing the *Aeromonas* host *luxR* locus are available at NCBI (accession no. NZ_CDBI01000063.1 and NZ_CDBI01000091.1, respectively). The genome sequence of the uncharacterized *Myoviridae* phage (Fig. S1A) is available at NCBI (accession no. MH622937.1) as “Myoviridae sp. isolate ctcg_2”. PHASTER analysis was performed at http://phaster.ca/ using the *A. popoffii* contig as the input (10).

### (viii) Data and software availability.

All experimental data that support the findings of this study are available from the corresponding author upon request.
