# Supplementary figures and images for: Phage-Encoded LuxR-Type Receptors Responsive to Host-Produced Bacterial Quorum-Sensing Autoinducers
Source: mBio. 2019 Apr 9;10(2):e00638-19. doi: 10.1128/mBio.00638-19 (PMC6456758; doi:10.1128/mBio.00638-19)

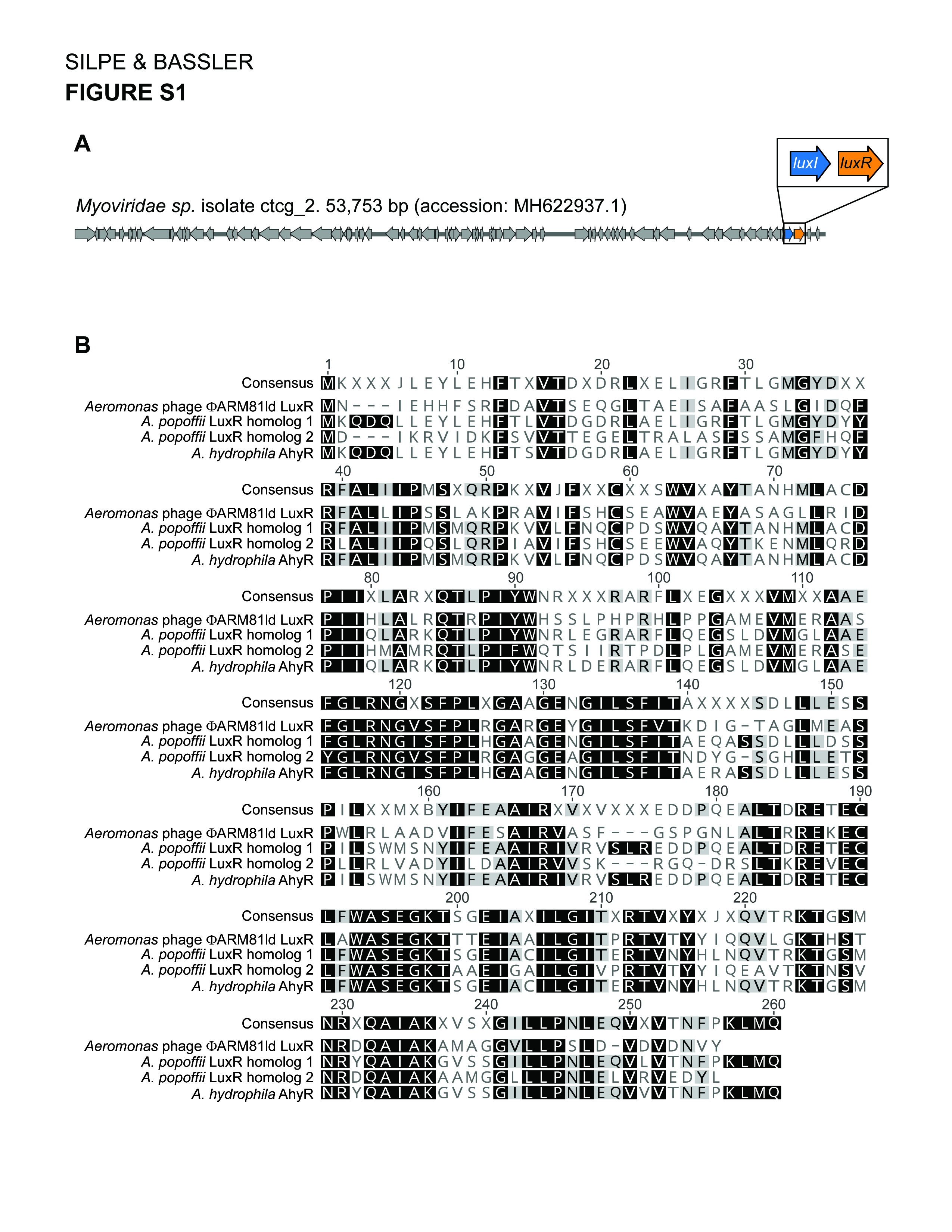

Supplement: FIG S1 [file mBio.00638-19-sf001.tif]

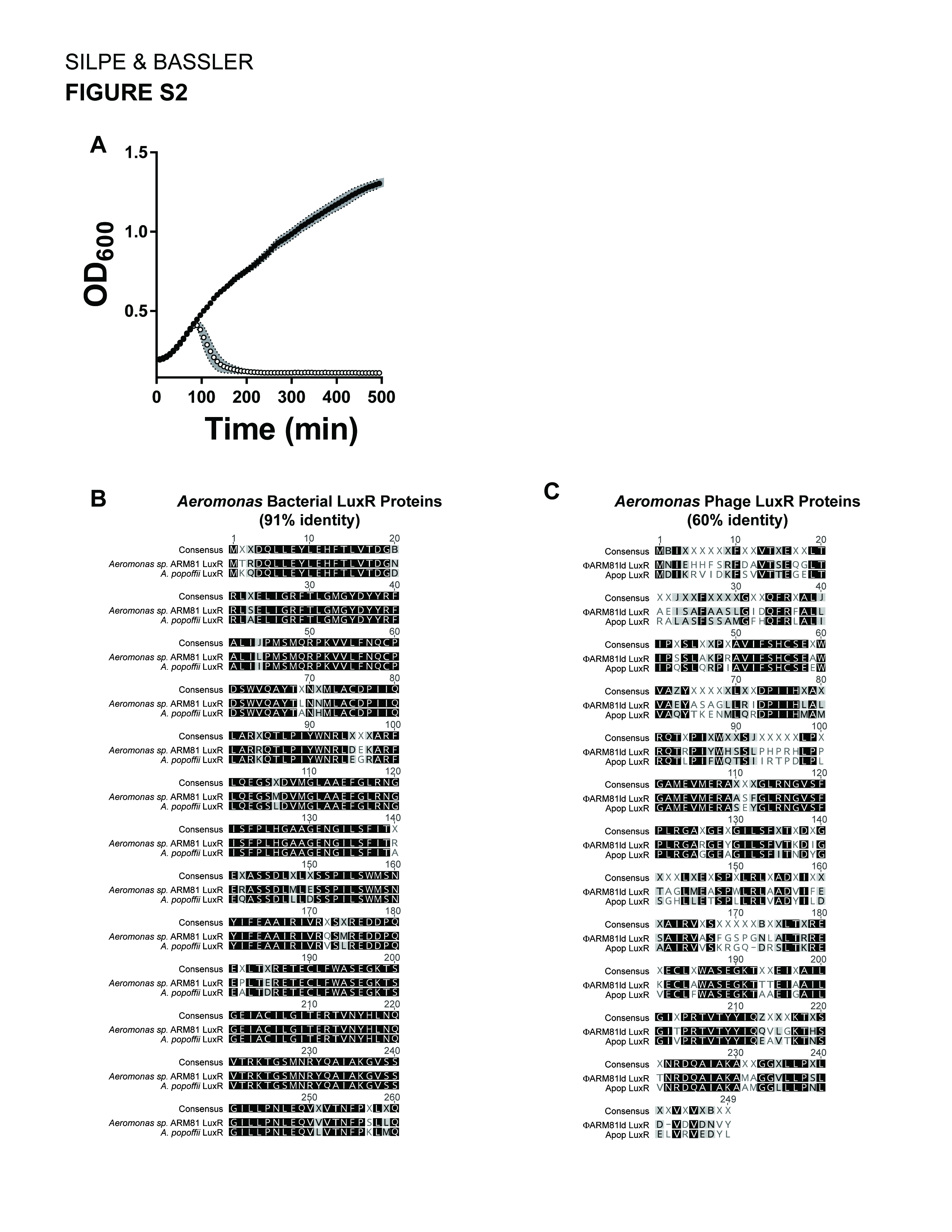

Supplement: FIG S2 [file mBio.00638-19-sf002.tif]

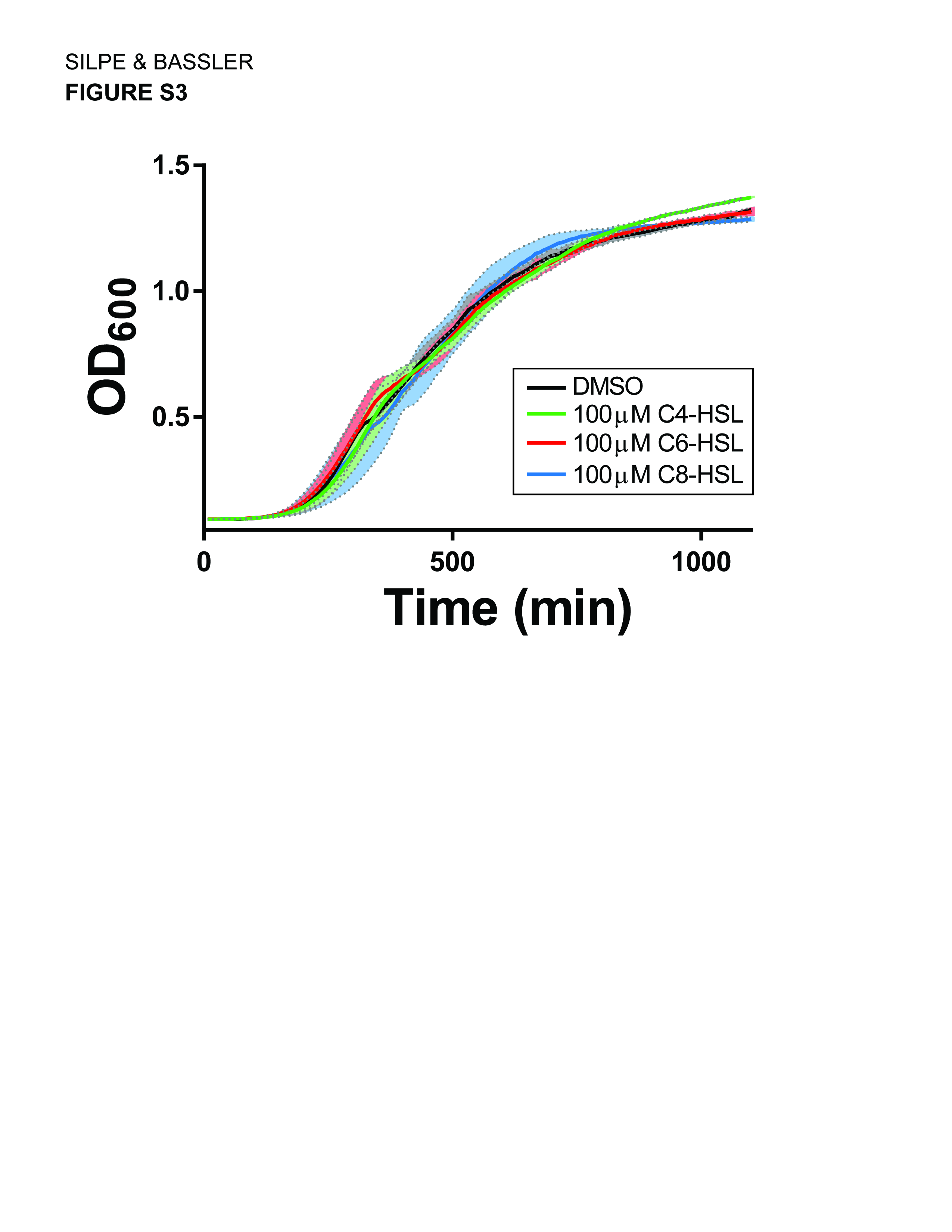

Supplement: FIG S3 [file mBio.00638-19-sf003.tif]
