# Supplementary material for: Phage-Encoded LuxR-Type Receptors Responsive to Host-Produced Bacterial Quorum-Sensing Autoinducers
Source: mBio. 2019 Apr 9;10(2):e00638-19. doi: 10.1128/mBio.00638-19 (PMC6456758; doi:10.1128/mBio.00638-19)
Supplement: TABLE S1 [file mBio.00638-19-st001.docx]

**Table S1: Bacterial strains used in this study**

| **Strain** | **Genotype** | **Reference** |
| --- | --- | --- |
| *E. coli* TOP10 | *F– mcrA Δ(mrr-hsdRMS-mcrBC) φ80lacZΔM15 ΔlacX74 recA1 araD139 Δ(ara-leu)7697 galU galK λ– rpsL(Str^R^) endA1 nupG* | Invitrogen |
| *E. coli* T7Express *lysY/I^q^* | *E. coli str. B, MiniF lysY lacIq(Cam^R^) / fhuA2 lacZ::T7 gene1 [lon] ompT gal sulA11 R(mcr-73::miniTn10-Tet^S^)2 [dcm] R(zgb-210::Tn10-Tet^S^) endA1 Δ(mcrC-mrr) 114::IS10* | NEB |
| *A. popoffii* CIP 105493 | Wild-type, LMG 17541; BAA-243; CIP 105493 | ATCC BAA-243 |
