# Supplementary material for: Phage-Encoded LuxR-Type Receptors Responsive to Host-Produced Bacterial Quorum-Sensing Autoinducers
Source: mBio. 2019 Apr 9;10(2):e00638-19. doi: 10.1128/mBio.00638-19 (PMC6456758; doi:10.1128/mBio.00638-19)
Supplement: TABLE S2 [file mBio.00638-19-st002.docx]

**Table S2: Plasmids used in this study**

| **#** | **Plasmid name (informal)** | **Plasmid ID** | **Strain ID (formal)** | **Relevant fragment** | **Marker, Origin** | **Source** |
| --- | --- | --- | --- | --- | --- | --- |
| 1 | pH6HTC-pT7*-HIS-HALO-cI_VP882_* | pJES-178 | JSS-1850 | *HIS-HALO-cI_VP882_* | Amp, pBR322 | This study |
| 2 | pH6HTC-pT7*-HIS-HALO-LuxR_ΦARM81ld_* | pJES-179 | JSS-1868 | *HIS-HALO-luxR_ΦARM81ld_* | Amp, pBR322 | This study |
| 3 | *repA_Apop_* | pJES-180 | JSS-1930 | *repA_Apop_* | Cm, Apop and oriR6ky | This study |
| 4 | pH6HTC-pT7*-HIS-HALO-LuxR_Apop_* | pJES-181 | JSS-1950 | *HIS-HALO-luxR_Apop_* | Amp, pBR322 | This study |
| 5 | pBAD*-RhlR* | pJP-2 | JP-117 / BB-0386 | *rhlR* | AmpR, pBR322 | (1) |
| 6 | P*rhlA-lux* | pJP-11 | JP-117 / BB-0386 | P*rhlA-lux* reporter | KanR, pSC101 | (1) |

**Supplemental references for Table S2.**

1. Paczkowski JE, Mukherjee S, McCready AR, Cong J-P, Aquino CJ, Kim H, Henke BR, Smith CD, Bassler BL. 2017. Flavonoids Suppress *Pseudomonas aeruginosa* Virulence through Allosteric Inhibition of Quorum-sensing Receptors. J Biol Chem 292:4064–4076.
