# Supplementary material for: Phage-Encoded LuxR-Type Receptors Responsive to Host-Produced Bacterial Quorum-Sensing Autoinducers
Source: mBio. 2019 Apr 9;10(2):e00638-19. doi: 10.1128/mBio.00638-19 (PMC6456758; doi:10.1128/mBio.00638-19)
Supplement: TABLE S3 [file mBio.00638-19-st003.docx]

| **Primer** | **Sequence (5' - 3')** | **5' Mod*** |
| --- | --- | --- |
| JSO-1268 | GAGCCAACCACTGAGGATCT |  |
| JSO-1435 | GACGTTACCAAAATTCATCATTAATTAACCTCCTG |  |
| JSO-1438 | GTTTTTTAATTAATTGGCCGATGATATGAACATAGAGCAC |  |
| JSO-1440 | GTAGACGTTGTCGACATCCACG | P' |
| JSO-1502 | GTTTTTTAATTAATGGACATCAAACGGGTTATCGATAAATTCA |  |
| JSO-1503 | CAGATAATCCTCTACTCTGACCAGCT | P' |
| JSO-1452 | CCTCGATCGTTTAATCCACTCGATAGA |  |
| JSO-1462 | AGAGCTATCAGGCTCATACACCC |  |
| JSO-0931 | CTGTCTCTTATACACATCTTCTAGAAGAAGCTTGGGATC | P' |
| JSO-0932 | CTGTCTCTTATACACATCTCTGTTGCATGGGCATAAAG | P' |
| JSO-1522 | AGCCGAGTCCGTTTACCGG |  |
| JSO-1514 | GACATCAAACGGGTTATCGATAAATTCAGC | P' |

**Table S3: Oligonucleotides and dsDNA used in this study**

| **dsDNA** | **Sequence (5' - 3')** |
| --- | --- |
| JSgblock-93 | GCCAACCACTGAGGATCTGTACTTTCAGAGCGATAACGCGGCCGATGATATGAACATAGAGCACCACTTCTCCCGCTTTGACGCGGTAACATCAGAACAAGGGCTGACAGCAGAGATTTCAGCGTTTGCAGCCAGCCTCGGGATTGACCAGTTCCGCTTCGCCTTGCTCATCCCCTCGTCACTGGCAAAGCCTCGGGCCGTCATTTTCAGCCACTGCAGTGAGGCTTGGGTGGCCGAATATGCCAGCGCTGGCTTGCTTCGAATCGACCCTATCATCCATCTAGCACTGCGTCAGACCCGCCCCATTTATTGGCACTCAAGCCTGCCTCATCCTCGGCACCTCCCCCCAGGGGCAATGGAGGTCATGGAGCGGGCTGCTTCTTTCGGCCTGCGCAACGGGGTGTCTTTTCCGCTGAGGGGGGCTCGAGGGGAGTATGGGATCCTGTCGTTCGTGACGAAGGACATCGGCACTGCGGGCTTGATGGAGGCCAGTCCCTGGCTTCGGCTGGCGGCTGACGTGATTTTTGAGTCGGCCATTCGGGTGGCATCGTTCGGAAGCCCCGGAAACCTGGCCCTGACCCGCCGCGAAAAGGAGTGCCTGGCGTGGGCCAGTGAGGGCAAGACGACCACCGAGATTGCCGCTATTCTTGGCATCACCCCCAGGACGGTGACCTATTACATTCAGCAGGTACTGGGGAAGACCCACAGCACGAACCGGGATCAGGCGATTGCCAAGGCGATGGCCGGCGGTGTGCTGCTTCCTAGCCTGGACGTGGATGTCGACAACGTCTACTGATTAATTAACCAATTCCTGCAGGATTTTGCGGCCGCTTGCT |

*indicates phosphorylation at the 5’ end
